# Supplementary material for: School-aged Schistosoma mansoni infection levels after long-term programmatic control show failure to meet control programme targets and evidence of a persistent hotspot: evaluation of the FibroScHot trial baseline data
Source: PLoS Negl Trop Dis. 2025 May 30;19(5):e0012708. doi: 10.1371/journal.pntd.0012708 (PMC12165415; doi:10.1371/journal.pntd.0012708)
Supplement: S1 Fig — N = 25 (52.1%) in Buhirigi and 26 (52%) in Kaiso answered “yes” to treating their water (χ2 < 0.001, p = 0.993). Shown are the percentages for how those individuals treated their water. Only answers with >5% of respondents in either community are displayed, with other methods grouped together. Chi-squared analysis of how water was treated: χ2 = 20.527, p < 0.001. (PDF) [file pntd.0012708.s001.pdf]

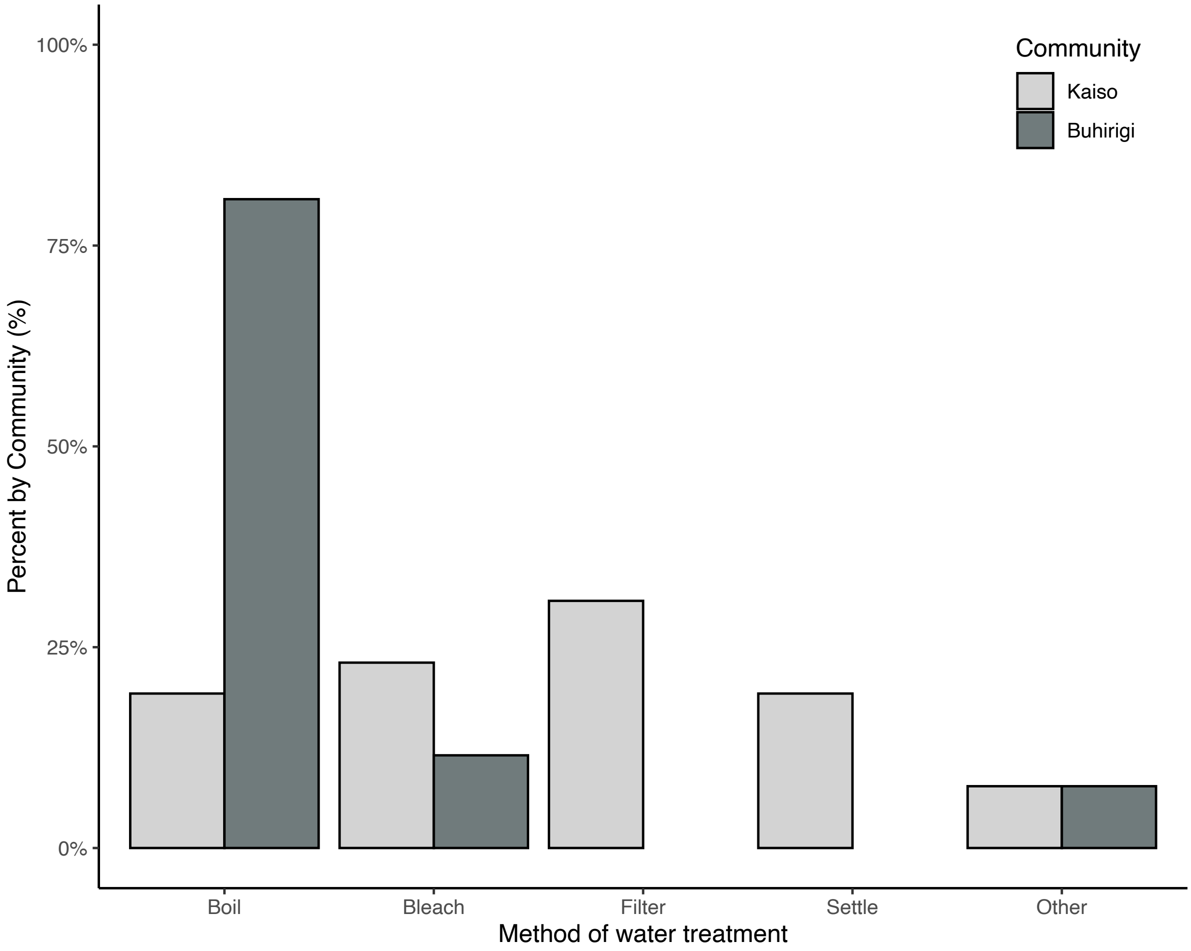

Figure S1: Reported ways of water treatment in Buhirigi and Kaiso communities. N=25 (52.1%) in Buhirigi and 26 (52%) in Kaiso answered "yes" to treating their water ($\chi^2 < 0.001$, $p = 0.993$). Shown are the percentages for how those individuals treated their water. Only answers with >5% of respondents in either community are displayed, with other methods grouped together. Chi-squared analysis of how water was treated: $\chi^2 = 20.527$, $p < 0.001$.
