# Supplementary material for: School-aged Schistosoma mansoni infection levels after long-term programmatic control show failure to meet control programme targets and evidence of a persistent hotspot: evaluation of the FibroScHot trial baseline data
Source: PLoS Negl Trop Dis. 2025 May 30;19(5):e0012708. doi: 10.1371/journal.pntd.0012708 (PMC12165415; doi:10.1371/journal.pntd.0012708)
Supplement: Attachment — Submitted filename: 6_Reply to comments.pdf [file pntd.0012708.s004.pdf]

We again thank the reviewers for their time taken. Please find below our point-by-point replies to their comments.

**Methods**

Reviewer #2: A few questions regarding the methods:

1. How were the two 'contrasting' communities (as mentioned in the introduction in line 103-104) selected for this study? You mention how the schools withing these two communities were selected, but it is unclear how the communities itself were selected.

*The selection process was solely that described for the schools and the further characteristics of the surrounding communities were not considered during the selection process. We have now added "two schools in " before "contrasting communities" in the last paragraph of the introduction to clarify the schools rather than their surrounding communities as the main unit of study.*

2. In the selection criteria you mention proximity to each other, does this mean schools had to be close together or far apart?

*In our study area description we have added "relatively close" to criteria number 4 to clarify that the schools had to be close rather than far apart.*

3. To be able to compare to the WHO definition you state that you only used the first stool sample for the analysis, does this mean a single slide from the first stool sample, or two slides from the first stool sample? Earlier in the same paragraph you state that two slides from each sample were prepared ('where possible'). It would be good to be specific on what was used for the analysis (i.e. one slide from a single stool sample).

*In our "Sample collection and processing" section we have changed this to " the **mean of the** egg counts obtained from the **two slides prepared from** the first stool sample" to clarify this (introduced text in bold).*

**Results**

Reviewer #2: Major comment regarding Table 1: in the 2016 monitoring exercise, what is the actual the S. mansoni prevalence? The table includes 2x 'negative'?

Please check the numbers in the table carefully: the prevalence numbers do not add up (16+33=49 for Buhirigi, and 34+31=65 for Kaiso?), as well as the intensity of infection numbers for Buhirigi (43+6=49?)

Also, be consistent in the number of decimals used (2 decimals for egg counts not necessary?)
The layout of the table needs improvement.

*We thank the reviewer for spotting the errors in table 1 in relation to the 2016 monitoring exercise. We apologise for these and have corrected them (Buhirigi should have been n=49 not 50; the number of those without detectable infections in Kaiso should have been 20 not 34, and the second "negative" has been replaced with "positive").*

*To improve the layout, we have changed the order of the columns in which the schools appear to: Kaiso, Buhirigi combined and then Buhirigi split by the year of sampling. This prevents empty cells directly after the row names for the 2016 monitoring exercise. We have reduced all prevalence and egg count data down to one decimal place (the exception to the one decimal place is the use of two for the standard error reported for age) and moved "epg" from the row label into the cells reporting the infection intensity, reducing the number of commas in the row label.*

**Conclusions**

Reviewer #2: The conclusions are supported by the data and the limitations are clearly described. It will be very interesting to see the follow-up data of the trial, whether or not increased frequency of treatment has a better effect on the (intensity of) infection in these affected communities.

*We thank the reviewer for these favourable comments.*

**Editorial and Data Presentation Modifications?**

**Summary and General Comments**

Reviewer #1: It is clear that modified approaches are required for SCH areas that are not responding to treatment. These areas in Uganda are a good case study. As the authors mention they have been receiving treatment for around 15 years and there is still significant infection.

Comments

- When considering the definition of hotspot, my biggest question is around what counts as baseline. In this paper the authors, understandably, use the 2016 monitoring exercise as the de facto baseline. But, as they also point out, there have been more than 15 years of treatment in these areas so the true underlying endemic baseline is likely to be much higher. Is there good data on what the levels of infection were originally, prior to any treatment? If not, are we in danger of using misleading figures?

    *We thank the reviewer for this pertinent comment. We do not hold pre-PC programme data for both our study villages, and whilst we acknowledge that "persistence" classification may differ if comparison with pre-PC data was possible, we also recognise that socio-environmental changes may have occurred over the last two decades influencing the transmission of S. mansoni in the area, making interpretation of any comparison with pre-PC data difficult. We have added a further paragraph to our discussion with the caveat that we will have captured the on-going impact of MDA rather than the immediate impact.*

- Relatedly, if an area has received 10/15 rounds of treatment and not reached control targets, can safely assume it's an area worthy of greater focus. The levels of infection remain high in both areas so by any sensible classification, both sites require additional interventions. Is there worth in suggesting a new definition for e.g. a 'zone of concern"? For example, after x years of treatment of at least y% coverage, the threshold has not been met? I'm worried we could get so caught up in whether somewhere meets the definition of hotspot that we miss important areas. And these are undoubtedly important areas.

    *We agree whole heartedly with the reviewer that one connotation of our results is that areas such as Buhirigi could be dismissed as not requiring additional interventions as they fail to meet the persistent hotspot criteria. We have used the reviewer's "zone of concern" phrase in our new discussion paragraph to highlight the need for ongoing operational research into optimal control in such areas. Given, however, that this manuscript concerns only the baseline findings of the FibroScHot programme our preference is not to make any recommendations here as we are yet to evaluate all our outcomes and hope to have a fuller understanding of ways in which to optimise control in areas like Buhirigi once we have.*

- It would be worth referencing the work that the trachoma community has done recently 'persistent' and 'recrudescent' areas and how they have developed modified strategies to reach them, e.g. 'more frequent than annual' treatment. There are similarities with the work being done here, and could be some good lessons. 'Informal consultation on end-game challenges for trachoma elimination' (https://iris.who.int/bitstream/handle/10665/363591/9789240048089-eng.pdf)

    *Whilst we agree that there are commonalities between the work of the trachoma community and what is discussed in our manuscript, the evidence that moderate as well as heavy S. mansoni infections cause poor health outcomes has led to significant debate as to the validity of S. mansoni control programme aims that utilise thresholds based upon heavy infections. Given this, we are reticent about making a direct comparison with the trachoma community approach of using failure to meet control programme aims as the basis for alternative approaches to control. Instead we hope our added discussion section on the need for ongoing operational research for "zones of concern" suffices.*

- Conclusion – you say that integrated approaches (including snail control and WASH) will likely be crucial for attainment of elimination. But the paper doesn't give any data to support that. It's possible (although I think the evidence base is equivocal) but not explored here.

*On reflection, we agree with the reviewer that our original phrasing is a little strong as we did not evaluate integrated approaches. We have modified the sentence to temper the conclusion in line with our findings. It now reads: "The intermediate snail host collections and the WASH questionnaire results show that there is scope for integrated approaches to be employed towards the attainment of elimination as a public health problem in these communities."*

- Check numbers and percentages in Table 1 – especially for top S.mansoni prevalence rows. Te numbers do not add up for Kaiso (34+31 don't equal 51). 'Negative' has been repeated.

*Please see reply to reviewer 2 under results section above.*

- Prevalence categories – would it make more sense to have three categories: Uninfected, light/moderate, heavy? Or even four: Uninfected, light, moderate, heavy. Rather than collapsing uninfected with light and moderate. A real difference between 0 and 399 epg!

*As requested the prevalence categories have been changed in table 1. As the hotspot definition uses prevalence of no/yes infected, this has been kept as a category within the table. The uninfected/light/moderate classification has now been replaced with the prevalence of light and moderately infected and the contribution of those uninfected removed from these numbers to prevent duplication with the prevalence no/yes data.*

- Sample size – how useful is the 2016 baseline data if there are only 50 children in two schools? Is that sufficient for study? Relatedly, how useful is the Bhugiri baseline parasitology data if there was a year of treatment between them?

*The 2016 monitoring was conducted in line with the WHO evaluation guidelines for national control programme managers. We have made an addition to the discussion to address how this relatively small sampling number may carry a risk of not accurately reflecting the true infection levels within the SAC population to the first paragraph of our discussion.*

*The split baseline was a consequence of the COVID-19 pandemic and out of our control. In our material and methods we describe fully the context of our data collection in relation to the previous PC round implemented. Given that there was a failure to meet control thresholds in Buhirigi for the data collection in 2020 and 2021, we believe that there is worth in this data, and throughout our results and discussion we have been diligent in taking the split baseline into account, presenting the data both as a whole for Buhirigi and stratified by year. As participants continued in the trial regardless of baseline sampling year we are of the opinion that is it essential that the information remains as presented, as it allows the schistosome control field to interpret both the findings presented in the current manuscript on hotspot classification and the trial results once they are published.*

- Increase in infections in Kaiso is striking – both prevalence and intensity – what do you think the reasons are for this?

*Given the parallels between the 2011 ESPEN entry for Kaiso, which also reflects several years worth of MDA, and the baseline findings in 2021, it could be that the 2016 survey underestimated the infection prevalence and infection intensity in Kaiso. This is pertinent to the comment above regarding whether 50 individuals is sufficient as an evaluation sample size and it is within the context of the Kaiso findings that we have added the discussion regarding true infection levels described above.*

Reviewer #2: I read the manuscript with great interest. The FibroScHot trial is highly relevant, and in this first paper the authors describe the baseline findings and discuss whether the study sites should be considered as a hotspot based on WHO definitions and how this can influence the analysis of the trial results.

*We thank the reviewer for these comments.*
